# Heritable forms of hypertension

**DOI:** 10.1007/s00467-007-0537-8

**Published:** 2009-10-01

**Authors:** V. Matti Vehaskari

**Affiliations:** Department of Pediatrics, Louisiana State University Health Sciences Center, 200 Henry Clay Avenue, New Orleans, LA 70118 USA

**Keywords:** Plasma renin activity, Aldosterone, Mineralocorticoid, Na transport, Epithelial Na channel (ENaC), NaCl cotransporter (NCC)

## Abstract

Among the causes of secondary hypertension are a group of disorders with a Mendelian inheritance pattern. Recent advances in molecular biology have unveiled the pathogenesis of hypertension in many of these conditions. Remarkably, the mechanism in every case has proved to be upregulation of sodium (Na) reabsorption in the distal nephron, with accompanying expansion of extracellular volume. In one group, the mutations involve the Na-transport machinery in distal tubule cells themselves: the distal convoluted tubule (DCT) cell and the principal cell of the collecting duct. Examples include Liddle’s syndrome, with an activating mutation of epithelial Na channel (ENaC); two types of Gordon’s syndrome, with mutations in two regulatory kinases [with no lysine (K) serine/threonine protein kinases (WNK)1 or WNK4]; and apparent mineralocorticoid excess (AME), with an inactivating mutation in the glucocorticoid-metabolizing 11β-hydroxysteroid dehydrogenase type 2 enzyme (11HD2). In another group, abnormal adrenal steroid production leads to inappropriate stimulation of the mineralocorticoid receptor (MR) in the distal nephron. The pathophysiology may involve inappropriate production of aldosterone [in glucocorticoid-remediable aldosteronism (GRA) and familial hyperaldosteronism type II (FH II)], of cortisol (in familial glucocorticoid resistance), or of other steroid metabolites (in congenital adrenal hyperplasia and GRA). In contrast to earlier beliefs, hypertension in many of the inherited disorders may be mild, and electrolyte and acid-base abnormalities are often not present. Monogenic hypertension should therefore enter the differential diagnosis of any child or adolescent with hypertension. Plasma renin activity (PRA) is the appropriate screening tool for all types of inherited hypertension.

## Introduction

It has long been recognized that hypertension in children, in contrast to adults, is often secondary to a definable underlying etiology. Pediatricians have been taught to look for primary causes such as kidney disease, renovascular hypertension, coarctation of the aorta, pheochromocytoma, hyperthyroidism, and others. Even after the more obvious causes have been ruled out, there are a few children in whom secondary hypertension is strongly suspected but the conventional search for a primary etiology is unsuccessful. A number of these children have hypertension due to single gene mutation inherited in Mendelian fashion. Progress in genetics and molecular biology during the past decade has greatly advanced our understanding of the pathogenetic mechanisms of these conditions. Some of these familial disorders, once considered rare curiosities, are proving to be common enough to be included in the differential diagnosis of any hypertensive child. This review discusses the group of hypertensive disorders caused by a single gene mutation in which recent research, much of it done in the laboratory of Richard Lifton at Yale University, has unveiled the mechanism leading to increased blood pressure. Other hereditary conditions such as familial pheochromocytoma, Williams syndrome, neurofibromatosis, and polycystic kidney disease are beyond the scope of this discussion.

Two features are shared by all types of monogenic hypertension in which the mechanism has been solved. First, the functional consequence of the abnormal gene in each case is increased sodium (Na) transport in the distal nephron. Second, plasma renin activity is invariably suppressed. The inescapable conclusion is that the hypertension is due to expanded extracellular volume resulting from inappropriate Na reabsorption by the distal tubule. This conclusion is consistent with the well-established role of the distal nephron in physiologic regulation of extracellular volume and with the hypothesis, initially formulated by Arthur Guyton, that failure of the renal Na-natriuresis mechanism to regulate Na and volume balance is part of any sustained hypertension, including essential hypertension [1, 2]. Because the unraveling of the cellular mechanisms of inherited hypertension has led to new insights into normal physiology of the distal nephron, an overview of distal nephron Na transport is briefly presented before the different disorders are discussed.

## Na transport in distal nephron

**Fig. 1:**
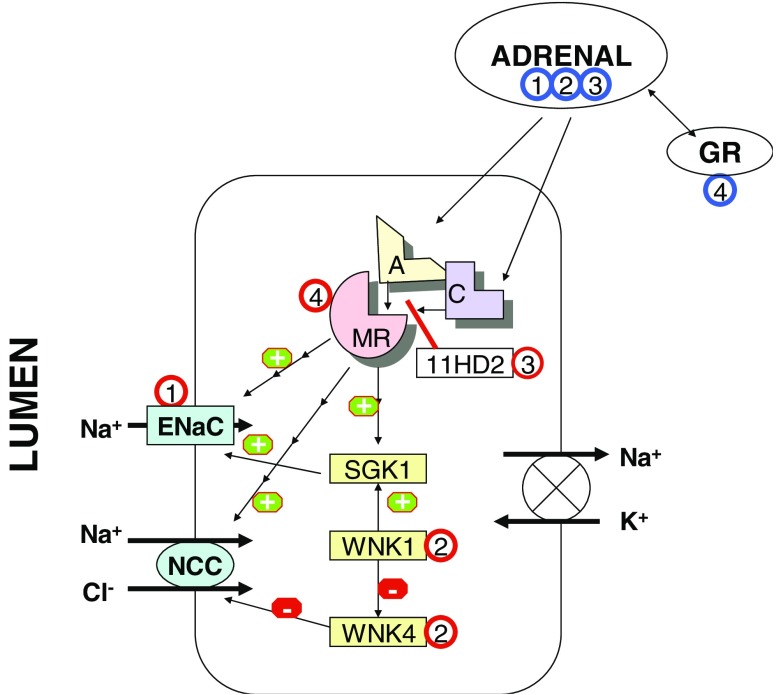
Simplified diagram of distal nephron sodium (Na)-transporting cell [distal convoluted tubule (DCT) cell and collecting duct principle cell (PC)]. The predominant apical Na transporter in the DCT cell is the thiazide-sensitive sodium chloride cotransporter (NCC) and in the collecting duct PC amiloride-sensitive epithelial sodium channel (ENaC), but some overlap exists between the cell types. Both are shown in the same cell in the diagram. For clarity, other transporters such as the potassium (K) channels are omitted.* A* aldosterone,* C* cortisol,* GR* peripheral tissue glucocorticoid receptor. Activation and suppression are indicated by* circled + * and * circled − *, respectively. Location of mutated proteins is indicated by* circled numbers* corresponding to the numbers in the text;* red circles* denote primary distal nephron disorders;* blue circles* denote adrenal and peripheral glucocorticoid receptor disorders

The two main Na-transporting cell types in the distal nephron are the distal convoluted tubule (DCT) cell and the majority cell in the collecting duct, the collecting-duct principal cell (PC). In addition, a transitional cell type may be present in the late DCT (connecting tubule), with features of both cell types. The basic configuration of the two cell types, illustrated in Fig. 1, is similar. The target of regulation and the rate-limiting step in the transepithelial Na transport is Na entry down its concentration gradient into the cell through the apical (luminal) Na transporter. In the DCT cell, the luminal Na transporter is the thiazide-sensitive sodium chloride (NaCl) cotransporter (NCC); in the principal cell, it is the amiloride-sensitive epithelial Na channel (ENaC). Na is actively extruded from the cell by the basolateral (contraluminal) sodium-potassium adenosine triphosphate (Na/K-ATPase), which is responsible for maintaining the low cytoplasmic Na concentration. K secretion is accomplished through the renal outer medulla K channel (ROMK) in the apical membrane, but K channels are also present in the basolateral membrane. Under most circumstances, the DCT is believed to exhibit modest K secretion, whereas K secretion in the collecting duct can achieve high rates depending on homeostatic needs. Na absorption from the lumen maintains the lumen-negative transmembrane potential that contributes to the driving force for K and proton secretion in the collecting duct; excessive Na reabsorption may lead to hypokalemia and metabolic alkalosis.

Regulation of Na transport in the distal nephron is incompletely understood. The best documented mechanism is the regulation by mineralocorticoids. Binding of a ligand (aldosterone) to the mineralocorticoid receptor (MR) in the target cells leads, via genomic and nongenomic signaling systems including serum- and glucocorticoid-inducible protein kinase 1 (SGK1), to increased activity of the apical Na transporter. The MR itself does not discriminate between aldosterone and cortisol; the mineralocorticoid-specificity is conferred by the cytoplasmic 11β-hydroxysteroid dehydrogenase type 2 enzyme (11HD2), which rapidly converts cortisol to inactive metabolites and thus protects the MR from “illicit” occupation by cortisol. The importance of the enzyme becomes clear when one considers that the ratio of aldosterone to cortisol in circulation is 1:100–1,000. Although traditionally the collecting duct PC has been considered the target of aldosterone, it is now recognized that the DCT cell (and the connecting tubule cell) is also mineralocorticoid responsive [3]. Therefore, Na reabsorption is under mineralocorticoid control in both the DCT and the collecting duct.

Recent advances in the molecular biology of Gordon’s syndrome (see below) have uncovered previously unknown distal nephron regulatory elements [4, 5]. Two kinases, with no lysine (K) serine/threonine protein kinase (WNK)1 and WNK4, expressed in the distal nephron cells, participate in the regulation of Na and K transport. WNK4 phosphorylates NCC, which prevents incorporation of the transporter into the apical membrane. By this mechanism, WNK4 exerts a tonic baseline suppression on NCC activity, which explains why interference with WNK4 can lead to augmented Na transport. WNK1 has no direct effect on NCC, but it modifies Na transport through inhibition of WNK4. In addition, WNK1 was recently been shown to activate ENaC through activation of SGK1 [6]. Moreover, both WNK1 and WNK4 appear to influence K transport in the DCT and the collecting duct by mechanisms separate from Na transport regulation, but the picture is still far from complete [7, 8].

It is clear from the above physiologic considerations that there is a large number of candidate genes whose mutations may explain Na transport abnormalities in the distal nephron. Only those resulting in upregulated transport and hypertension are discussed below. The known disorders can be divided into (1) primary disorders of the distal nephron, and (2) primary adrenal disorders.

## Distal nephron disorders

### 1. Liddle’s syndrome

Collecting-duct Na transport ultimately depends on the activity of ENaC, which consists of three subunits encoded for by separate genes. ENaC is also present in the terminal portion of DCT. Several mutations in the β and γ subunits have been described that result in increased ENaC-mediated Na flux [9, 10]. The channel activity is, in part, regulated by recycling of the transporter between the apical membrane and subapical vesicles. It has been shown that the mutations truncate or modify the cytoplasmic tail of the transmembrane protein, deleting the binding site necessary for the retrieval of the channel for degradation or recycling [11]. Therefore, the number of functioning channels in the membrane is increased. In addition, the mutations may render the membrane-bound subunit proteins susceptible to proteolytic cleavage, which further enhances the channel activity by increasing its open probability [12].

As expected for activating mutations, the inheritance pattern is autosomal dominant. Liddle’s syndrome may be the most common type of monogenic hypertension, and it has been reported in many ethnic populations, including Caucasians, Asians, and blacks. Hypertension frequently begins in childhood but may be asymptomatic and escape detection until early adulthood. The presence of hypokalemia and mild metabolic alkalosis is variable. Plasma renin activity (PRA) and plasma aldosterone levels are markedly suppressed.

Treatment consists primarily of a low-salt diet and an agent that directly inhibits ENaC, such as amiloride or triamterene. It is important to recognize that MR antagonists are without effect. Strict adherence to low-salt diet often removes the need for other antihypertensive medications, but some patients, especially after long-standing hypertension, may require nonspecific treatment with other antihypertensives such as calcium (Ca)-channel blockers. Untreated individuals are at high risk for cardiovascular morbidity and mortality.

### 2. Gordon’s syndrome (familial hyperkalemic hypertension, pseudohypoaldosteronism type II)

The phenotypic features of Gordon’s syndrome are hyperkalemia, mild metabolic acidosis, and hypertension. Because the abnormalities respond to low-dose thiazide treatment, the syndrome was linked to abnormal function of the thiazide-sensitive nephron segment, the DCT, long before any gene mutations were discovered. It is now clear that mutations in at least four different genes may cause Gordon’s syndrome, although only two have been identified. In contrast to expectations, no activating mutations in the NCC itself have been found despite the clinical evidence that Na reabsorption in DCT is increased. Instead, mutations in the newly discovered kinases WNK1 and WNK4 have been reported in some patients, and further research has revealed the likely underlying mechanisms [4, 5, 8]. The culprit in some families is an inactivating mutation in WNK4 that releases NCC from the tonic suppression by WNK4, leading to constitutively increased Na reabsorption in DCT (Fig. 1). In contrast, in regard to their effect on ROMK, the same mutations may increase the activity of WNK4, further enhancing the suppressive effect of WNK4 on ROMK and explaining the hyperkalemia [7].

In the second type of Gordon’s syndrome, a large intronic mutation causes an increase in WNK1 expression. Because the indirect physiologic effect of WNK1 on NCC is activation (Fig. 1), the mutation causes upregulation of Na transport [4, 5]. WNK1 may also activate ENaC via SGK1 [6]. The recent demonstration of two different WNK1 isoforms in the kidney, with opposing effects on ROMK, complicates the understanding of the K retention in WNK1 mutations [8]. At least two additional monogenic types of Gordon’s syndrome, apparently not involving WNK kinases, have been described, but the underlying gene defects are unknown [13, 14].

Gordon’s syndrome is inherited as an autosomal dominant trait. This is unusual but not unique for a loss-of-function mutation such as the WNK4 mutation. There are other examples of loss-of-function in one allele resulting in disease. Whether the clinical features differ between different genotypes of Gordon’s syndrome is unknown. The metabolic abnormalities hyperkalemia and hyperchloremic metabolic acidosis tend to precede the onset of hypertension, and hypertension often does not manifest until adult life [15, 16]. In fact, Spitzer-Weinstein syndrome in children, consisting of hyperkalemia, metabolic acidosis, and growth failure but not hypertension, is now believed to be an early presentation of Gordon’s syndrome [7, 17–19]. Thus, the presentation may resemble that of type IV renal tubular acidosis.

Diagnostic features, in addition to hyperkalemia and acidosis, include suppression of plasma renin activity and normal or high aldosterone level. The high aldosterone level is explainable by direct stimulation of aldosterone secretion by high plasma K levels despite the increase in extracellular volume. Plasma K and acid-base status are not invariably abnormal. Glomerular filtration rate is normal in contrast to many forms of type IV renal tubular acidosis. Hypercalciuria is present in many if not all patients with Gordon’s syndrome [16, 20]. Both hypertension and metabolic abnormalities typically respond to low-dose thiazide diuretics.

### 3. Apparent mineralocorticoid excess (AME)

Apparent mineralocorticoid excess (AME) is a rare autosomal recessive disorder consisting of an inactivating mutation of the 11HD2 enzyme [21]. This allows cortisol to occupy and activate the MR (Fig. 1). The typical features are unregulated stimulation of Na reabsorption, K secretion, and hydrogen (H) secretion. This is accompanied by renal concentrating defect, most likely due to chronic hypokalemia, and sometimes by hypercalciuria and nephrocalcinosis, the mechanisms of which is unclear [22]. As a result of lack of 11HD2 activity, the conversion of cortisol to cortisone is impaired, resulting in an abnormal ratio of cortisol metabolites [such as tetrahydrocortisol (THF) and allotetrahydrocortisol (5αTHF)] to cortisone metabolites [such as tetrahydrocortisone (THE)] in the urine.

AME has been reported from all parts of the world and in many ethnic groups, including Caucasians, blacks, Asians, and American Indians. The disease was earlier believed to invariably present in early childhood, with a severe phenotype including low birth weight, failure to thrive, and hypokalemic metabolic alkalosis. These patients frequently exhibit end-organ damage in the heart, the retina, and the central nervous system, and the mortality rate in untreated patients is high [23, 24]. More recently, however, milder phenotypes have been described, some presenting with hypertension in adulthood without electrolyte abnormalities [22, 25, 26]. The milder phenotypes are associated with mutations causing only partial inactivation of 11HD2 [22, 26].

Diagnosis should be suspected in any low renin, low aldosterone hypertension with signs of mineralocorticoid excess. Traditionally, the diagnosis has been confirmed by urine steroid profile, especially by the ratio of THF + 5αTHF to THE in a 24-h urine collection. However, in patients with mild disease, the ratio may not be abnormal, making diagnosis difficult except by genetic testing.

MR antagonists, K supplementation, and dietary Na restriction are the mainstay of treatment of AME. The more specific MR antagonist eplerenone, instead of spironolactone, can be used to minimize side effects. In addition, amiloride may be added to help conserve K. Thiazide diuretics have been used to ameliorate hypercalciuria.

### 4. Hypertension exacerbated by pregnancy

Geller and coworkers recently described a new genetic hypertension disorder called “hypertension exacerbated by pregnancy” [27]. The term is slightly misleading because the disorder is not limited to females; in fact, the proband was a 15-year-old male. The affected individuals have an activating mutation of MR, resulting in constitutively stimulated Na reabsorption (Fig. 1). Hypertension is present in nonpregnant patients and may be severe, but the characteristic feature is marked worsening during pregnancy. The proposed explanation is that the mutation renders the receptor sensitive to nonmineralocorticoid steroids such as progesterone, the level of which rises 100-fold during pregnancy. Notably, the mutated receptor can also be activated by spironolactone.

The inheritance pattern is autosomal dominant. The disorder is mostly of theoretical interest, because the estimated frequency of the precise mutation required for intrinsic receptor activation is so low that, at most, a few pedigrees are predicted to exist (D. Geller, personal communication). Treatment consists of salt restriction and thiazide diuretics or ENaC antagonists; MR receptor antagonists are contraindicated.

## Adrenal disorders

### 1. Glucocorticoid-remediable aldosteronism [(GRA) familial hyperaldosteronism type I]

The adrenal cortex synthesizes aldosterone in the zona glomerulosa under the control of angiotensin II and cortisol in the zona fasciculata under the control of the adrenocorticotropic hormone (ACTH). The key enzymes in the synthetic pathways are aldosterone synthase and 11β-hydroxylase, respectively. The corresponding genes lie adjacent to each other on chromosome 8. GRA is the result of unequal crossing over between the two genes. The hybrid gene possesses an ACTH-responsive promoter element and an aldosterone synthase coding region, resulting in ACTH-stimulated aldosterone production, independently of renin and of Na balance requirements [28–30]. In addition, two other steroids with mineralocorticoid activity, 18-oxocortisol and 18-hydroxycortisol, are produced from cortisol in the zona fasciculata by aldosterone synthase in GRA. The high levels of circulating mineralocorticoids activate distal nephron MR and upregulate Na reabsorption and K secretion.

The inheritance pattern of GRA is autosomal dominant. Different pedigrees exhibit different crossover patterns of the hybrid gene, suggesting that the mutations arose independently in each pedigree [31]. Notably, GRA has not been found in blacks.

Most affected individuals develop hypertension during childhood or even in infancy [5] and exhibit significant cardiovascular morbidity [32, 33], but milder phenotypes and even normotension have been described [31, 32, 34]. A number of patients have been first diagnosed with hypertension in adolescence [35]. Phenotype severity may vary within the same family. GRA patients appear to be particularly at risk for cerebral aneurysms and intracranial bleeding, and magnetic resonance angiographic (MRA) screening beginning at puberty has been recommended [36].

Mild hypokalemia is present in less than half of affected individuals, and a few exhibit mild metabolic alkalosis [31, 32, 35]. PRA is suppressed, but plasma aldosterone concentration is not always outside the reference range. Ratio of plasma aldosterone (ng/dl) to plasma renin activity (ng/ml/h) of over 30 (normal < 20) is highly suggestive of primary aldosteronism. Dexamethasone suppression test, urine steroid profiles (increased 18-oxocortisol), adrenal imaging, and adrenal-vein sampling have traditionally been used to distinguish GRA from other types of primary aldosteronism, but the availability of reliable genetic testing for the chimeric gene has eliminated the need for more cumbersome evaluation.

Treatment of GRA consists of glucocorticoids to suppress ACTH-stimulated mineralocorticoid production and/or of MR antagonists. Conventional high-dose glucocorticoid treatment is unnecessary. It is now clear that blood pressure can be controlled without total normalization of biochemical parameters using low-dose treatment. For adults, dexamethasone dose of 0.125–0.24 mg daily or prednisolone dose of 2.5–5 mg daily appears sufficient [33]. MR antagonist treatment is an alternative, either alone or in combination with low-dose glucocorticoid. The ENaC antagonists amiloride or triamterene can be used as an adjunctive therapy.

### 2. Familial hyperaldosteronism type II (FH II)

Familial hyperaldosteronism type II (FH II) is similar to GRA (FH I) in that excess mineralocorticoid production is responsible for the development of hypertension, but the hypertension is not suppressible by dexamethasone. Autosomal dominant inheritance suggests that FH II is due to a single gene mutation and, although the gene has not been identified, the locus has recently been narrowed to a band on chromosome 7 [37]. Many patients with FH II have a family history of adrenal hyperplasia or adenoma, which suggests that a growth factor may be involved.

Widened criteria for screening have revealed FH II to be more common than previously believed, and it may be the most common inherited type of hypertension in adults [38]. However, although renin-aldosterone abnormalities have been reported in affected adolescents [39], hypertension due to FH II typically does not manifest until adulthood. FH II is clinically and biochemically indistinguishable from noninherited primary aldosteronism, and currently, the diagnosis can be only confirmed by positive family history until genetic detection is available.

### 3. Congenital adrenal hyperplasia

In two subtypes of congenital adrenal hyperplasia, there is excessive accumulation of intermediate products with mineralocorticoid activity. Defects in 11-hydroxylase and 17-hydroxylase result in overproduction of 21-hydroxylated steroids, which activate MR. In addition to having other endocrine manifestations such as genital ambiguity, these patients are hypertensive due to Na retention mediated by the distal nephron. The diagnosis is made by clinical presentation and by analysis of plasma or urine steroid profiles. The hypertension responds to treatment with an MR antagonist. Note that the most common type of congenital adrenal hyperplasia, 21-hydroxylase deficiency, is an Na-losing state and does not cause hypertension.

### 4. Familial glucocorticoid resistance

High cortisol availability may overwhelm the capacity of the 11HD2 enzyme to metabolize it, leading to activation of the MR by cortisol and to excessive Na reabsorption in the distal nephron. This is the case in families with an inherited defect in the glucocorticoid receptor, rendering it partially unresponsive to cortisol and with consequent feedback overproduction of cortisol and androgens [40]. Inheritance pattern differs between different mutations [41]. The severity of associated features and the presence of hypertension depend on the degree of receptor impairment. Mutations causing complete receptor inactivation are not compatible with life. Because of the glucocorticoid resistance, the patients are not Cushingoid. Diagnosis of this rare disorder rests of documentation of markedly elevated plasma cortisol levels. The hypertension responds to MR blockade.

## Evaluation for inherited hypertension

When should a child be evaluated for possible inherited hypertension? Although the true prevalence of this group of disorders is not known, many of them, such as Liddle’s syndrome, are more common than previously suspected. Inheritance pattern, if clear, may provide clues (Table 1), but family history is often misleading because of the high prevalence of essential hypertension in adults and even adolescents. The conventional wisdom that hereditary conditions should be considered in children with severe hypertension or electrolyte abnormalities is only partially true. Whereas a child with severe hypertension is more likely to have one of the inherited disorders, it is now clear that many individuals with inherited hypertension have only mild to moderate hypertension, and electrolyte abnormalities may be absent (Table 1).

**Table 1:** Features of inherited hypertension

	Inheritance pattern	Age	K	PRA	Aldo	Aldo:PRA	GC resp.	MR-A resp.	Rx
Liddle’s	AD	C,A	N or ↓	↓	↓		–	–	A,Tr
Gordon’s	AD	A (C)	N or ↑	↓	N or ↑	↑	–	–	T
AME	AR	I,C,A	↓ (N)	↓	↓		–	+	MR-A
H-P	AD	C,A	N or ↓	↓	↓		–	reversed	A,Tr,T
GRA	AD	I,C	N or ↓	↓	↑ (N)	↑	+	+	G,A,Tr
FH II	AD	A	N or ↓	↓	↑	↑	–	+	MR-A
CAH	AR	I	N or ↓	↓	↓		–	+	MR-A
FGR	AR/AD	I	N or ↓	↓	↓		–	+	MR-A

*AME* apparent mineralocorticoid excess,* H-P* hypertension exacerbated by pregnancy,* GRA* glucocorticoid-remediable aldosteronism,* FH II* familial hyperaldosteronism type II,* CAH* congenital adrenal hyperplasia with 11- or 17-hydroxylase deficiency,* FGR* familial glucocorticoid resistance, * AD* autosomal dominant,* AR* autosomal recessive,* Age* typical age at presentation,* I* infancy,* C* childhood,* A* adulthood,* K* potassium,* N* normal,* ↓* decreased,* ↑* increased,* PRA* plasma renin activity,* Aldo* aldosterone,* Aldo:PRA* ratio of aldosterone to PRA (>30 diagnostic if Aldo. in ng/dl, PRA in ng/ml/h),* GC resp.* response to glucocorticoids,* –* negative,* +* positive,* MR-A resp*. response to mineralocorticoid receptor antagonists, *Rx* treatment, * A* amiloride,* Tr* triamterene,* T* thiazides

Hyperkalemia is frequently but not always present in Gordon’s syndrome; hypokalemia is present in less than 50% of GRA and often but not consistently present in AME and Liddle’s syndrome. Mild metabolic alkalosis may infrequently accompany all except Gordon’s syndrome, which is associated with metabolic acidosis. Importantly, because all patients must eventually develop a neutral, steady-state electrolyte balance despite initial expansion of extracellular volume, urine Na and K excretion cannot be used for diagnosis. Therefore, whereas clinical presentation and routine electrolyte values may raise a suspicion of inherited hypertension, they cannot be relied on to rule out these disorders.

An easy and useful screening tool is PRA, the inclusion of which in the initial evaluation of all hypertensive children is justified. This is preferably done before treatment because antihypertensive drugs may alter renin secretion. Because extracellular volume is expanded, resulting in suppression of the renin–angiotensin system in all of the disorders discussed in this review, they all share the feature of suppressed PRA. Ideally, PRA should be correlated with Na intake (measurable as 24-h Na excretion), but a random renin level is usually informative. If suppressed plasma renin activity is documented, the next step is to measure plasma aldosterone if not done initially. Suppressed plasma aldosterone is present in all inherited hypertension except primary aldosteronism (GRA, FH II) and Gordon’s syndrome and should therefore lead to a strong suspicion of AME or Liddle’s syndrome. In primary aldosteronism, the most discriminating test is the ratio of plasma aldosterone (ng/dl) to plasma renin activity (ng/ml/h); a value of greater that 30 indicates primary aldosteronism (Table 1).

Genetic testing for the mutations causing hereditary hypertension is increasingly becoming available. Information on testing for clinical purposes can be found at www.genetests.org. Final confirmation of GRA can nowadays reliably be made by direct documentation of the presence of the hybrid gene by Southern blotting instead of the traditional dexamethasone suppression test. However, in most of the other disorders, testing is based on direct DNA sequencing or on probing for known mutations and will not be able to detect previously unreported mutations. In some patients, only a presumptive diagnosis can be made based on clinical presentation, laboratory findings, and response to specific pharmacologic agents.

**Questions:**

(Answers appear following the reference list.)

1. A 20-year-old woman presents with headache and blood pressure of 185/100. She is on no medications. Her mother and 25-year-old brother have hypertension. Metabolic panel shows Na 142, Cl 115, K 5.6, CO_2_ 17. The treatment most likely to control her hypertension is:
  1. Ca-channel blocker
  2. Spironolactone
  3. Hydrochlorothiazide
  4. Amiloride
  5. Furosemide
2. A 3-year-old Caucasian boy is evaluated for hypertension. He has a 7-year-old brother with hypertension whose disease is poorly controlled with the combination of enalapril and nifedipine. The patient has normal electrolytes, plasma renin activity of 0.5 ng/ml per hour (normal for age 3.0–9.0), and plasma aldosterone concentration of 30 ng/dl (normal 3–35). The most likely diagnosis is:
  1. Liddle’s syndrome
  2. Gordon’s syndrome
  3. Renal artery stenosis
  4. Glucocorticoid-remediable aldosteronism
  5. Familial aldosteronism type II
3. A 2-year-old boy is referred for failure to thrive. His blood pressure is 145/95. Laboratory evaluation shows Na 139, K 3.0, Cl 90, CO_2_ 35, and plasma renin activity < 0.2 ng/ml/ per hour Renal ultrasound shows bilateral nephrocalcinosis. The most appropriate initial drug to treat hypertension is:
  1. Enalapril
  2. Spironolactone
  3. Hydrochlorothiazide
  4. Furosemide
  5. Aldosterone
4. Genetic testing can exclude the following conditions:
  1. Liddle’s syndrome
  2. Gordon’s syndrome
  3. Glucocorticoid-remediable aldosteronism
  4. Familial hyperaldosteronism type II
  5. Apparent mineralocorticoid excess syndrome
5. Inherited hypertension can be ruled out in a hypertensive 17-year-old if:
  1. He was normotensive at age 12
  2. His maximum blood pressure does not exceed 140/85
  3. His plasma renin activity is low
  4. His plasma renin activity is normal
  5. His plasma aldosterone concentration is normal
6. Which of the following treatments is not effective:
  1. Hydrochlorothiazide for Gordon’s syndrome
  2. Amiloride for Liddle’s syndrome
  3. Spironolactone for apparent mineralocorticoid excess
  4. Amiloride for apparent mineralocorticoid excess
  5. Spironolactone for Liddle’s syndrome

## Abbreviations

11HD2: 11β-hydroxysteroid dehydrogenase type 2 enzyme
AME: apparent mineralocorticoid excess
DCT: distal convoluted tubule
ENaC: amiloride-sensitive epithelial Na channel
FH II: familial hyperaldosteronism type II
GRA: glucocorticoid-remediable aldosteronism
MR: mineralocorticoid receptor
NCC: thiazide-sensitive NaCl-cotransporter
PC: collecting-duct principal cell
PRA: plasma renin activity
ROMK: renal outer medulla K channel
SGK1: serum- and glucocorticoid-inducible protein kinase 1
WNK1 and WNK4: With no lysine (K) serine/threonine protein kinases
